# Inhibition of *Staphylococcus aureus* biofilm formation by gurmarin, a plant-derived cyclic peptide

**DOI:** 10.3389/fcimb.2022.1017545

**Published:** 2022-10-04

**Authors:** Adeline W. Chang, Scot E. Dowd, Gordon Brackee, Joe A. Fralick, Govindsamy Vediyappan

**Affiliations:** ^1^ Division of Biology, Kansas State University, Manhattan, KS, United States; ^2^ MR DNA (Molecular Research), Shallowater, TX, United States; ^3^ Laboratory Animal Resources Center, Texas Tech University Health Sciences Center, Lubbock, TX, United States; ^4^ Immunology and Molecular Microbiology, Texas Tech University Health Sciences Center, Lubbock, TX, United States

**Keywords:** *staphylocccus aureus*, biofilm inhibitor, gurmarin, cyclic-peptide, *gymnema sylvestre*, knottin, rat implant, transcriptomic analyses

## Abstract

*Staphylococcus aureus (Sa)* is an opportunistic pathogen capable of causing various infections ranging from superficial skin infections to life-threatening severe diseases including pneumonia and sepsis. *Sa* produces biofilms readily on biotic and abiotic surfaces. Biofilm cells are embedded in a protective polysaccharide matrix and show an innate resistance to antibiotics, disinfectants, and clearance by host defenses. Additionally, biofilms serve as a source for systemic dissemination. Moreover, infections associated with biofilms may result in longer hospitalizations, a need for surgery, and may even result in death. Agents that inhibit the formation of biofilms and virulence without affecting bacterial growth to avoid the development of drug resistance could be useful for therapeutic purposes. In this regard, we identified and purified a small cyclic peptide, gurmarin, from a plant source that inhibited the formation of *Sa* biofilm under *in vitro* growth conditions without affecting the viability of the bacterium. The purified peptide showed a predicted molecular size of ~4.2 kDa on SDS-PAGE. Transcriptomic analysis of *Sa* biofilm treated with peptide showed 161 differentially affected genes at a 2-fold change, and some of them include upregulation of genes involved in oxidoreductases and downregulation of genes involved in transferases and hydrolases. To determine the inhibitory effect of the peptide against *Sa* biofilm formation and virulence *in vivo*, we used a rat-implant biofilm model. *Sa* infected implants with or without peptide were placed under the neck skin of rats for seven days. Implants treated with peptide showed a reduction of CFU and lack of edema and sepsis when compared to that of control animals without peptide. Taken together, gurmarin peptide blocks *Sa* biofilm formation *in vitro* and *in vivo* and can be further developed for therapeutic use.

## Introduction

*Staphylococcus aureus (Sa)* is an important human and animal bacterial pathogen responsible for a wide variety of conditions, ranging from superficial skin infections to serious life-threatening infections including pneumonia, endocarditis, osteomyelitis, septicemia, and toxin-mediated toxic shock syndrome in humans (Parsek and Singh, 2003; Brady et al., 2008), and chronic skin infections and mastitis in agricultural animals (Taponen and Pyorala, 2009). The threat of *Sa* infection is enhanced by the rise of specific antibiotic resistance mechanisms, including methicillin-resistant *S. aureus* (MRSA) and non-specific resistance such as biofilms. *Sa* produces a variety of cytotoxins and superantigens which allow the bacterium to evade host immune system and colonize successfully (Gotz, 2002; Otto, 2008). Many of these infections are associated with the formation of biofilms on either host tissues including skin wounds or implanted materials such as central venous catheters, urinary catheters, prosthetic heart valves, orthopedic implants, dialysis catheters. These biofilms are highly resistant to host defenses and antimicrobial therapies, and thus limit the available treatment options (Donlan and Costerton, 2002; Davies, 2003; Cassat et al., 2007; del Pozo and Patel, 2007). According to several estimates, microbial biofilms account for over 80% of all infections in the body including implanted medical devices (Davies, 2003; NIH, 2005; Vadyvaloo and Otto, 2005; Harraghy et al., 2006; Vestby et al., 2020).

*Sa* readily forms polymicrobial biofilms with other microbes including *Pseudomonas aeruginosa* and human fungal pathogen *Candida albicans*, and displays elevated resistance to antibiotics (Dowd et al., 2008; Harriott and Noverr, 2009). The pathogens produce extracellular polysaccharide materials, which play a number of roles in biofilms including sequestration of antimicrobial agents (Mah et al., 2003; Nett et al., 2007; Flemming and Wingender, 2010; Vediyappan et al., 2010). Because biofilms are resistant to host defenses and conventional antibiotics, and become a reservoir for persistent infection, novel agents that can prevent the development of biofilm and virulence will be useful for therapeutic purposes.

*Sa* biofilm formation involves the initial attachment of cells to surfaces and then grows into a multilayer of cells encased by extracellular polymeric materials. The matured biofilms then detach and disperse involving proteases and other enzymes. The biofilm growth is tightly controlled by several regulatory systems that integrate the physiology of the cells and the environmental signals. The accessory gene regulator (Agr) quorum sensing (QS) system, the cell density-dependent QS, is one of the best-studied regulatory systems in *Sa* biofilm formation (Novick and Geisinger, 2008; Kavanaugh and Horswill, 2016). The Agr QS system is controlled by the *agrBDCA* genes, which encode proteins necessary for the synthesis, transport, and signaling of QS autoinducing peptide (AIP). In addition to the Agr, there are several other regulatory systems, including SaeRS, SarA, Rot, SigB, LytSR, etc. are involved in *Sa* biofilm development (Schilcher and Horswill, 2020). In addition to the conventional antibiotics-mediated intervention, a broad range of approaches or inhibitors have been identified to prevent or eradicate *Sa* biofilms. These include small molecule inhibitors of the Agr QS and other virulence mechanisms, and biofilm-eradicating molecules, (Schilcher and Horswill, 2020; Song et al., 2022). Since *Sa* biofilm matrix contains polysaccharides, proteins, and DNA, compounds capable of dissolving matrix components (glycoside hydrolases, proteases, or DNAse) can disrupt established biofilms or prevent the formation of biofilm.

Molecules that inhibit biofilms but not the bacterial growth, to avoid selection pressure for resistance, has emerged as novel approach (Cegelski et al., 2008). Traditional medicinal plants could be a potential source to search for antibiofilm agents as these plants have been used for centuries in human health. Screening of medicinal plant-derived library of compounds identified *Gymnema sylvestre* (*Gs*) plant source that inhibited *Sa* biofilm growth without affecting bacterial growth or viability. Subsequent analysis of the bioactive fraction identified a polypeptide, gurmarin, as an inhibitory agent of *Sa* biofilm formation. *Gs* is a medicinal plant used for treating diabetes mellitus in Ayurveda traditional medicine (Porchezhian and Dobriyal, 2003; Kanetkar et al., 2007; Leach, 2007). This plant contains various small molecules with multiple pharmacological activities including anti-sweet, glucose uptake inhibitory, gut glycosidase inhibitory, and antimicrobial activities (Kanetkar et al., 2007; Vediyappan et al., 2013; Khan et al., 2019; Veerapandian and Vediyappan, 2019). The gurmarin polypeptide is 35 amino acids long and belongs to the knottin family of cyclic peptides, which are known to have diverse biological activities (Colgrave and Craik, 2004; Wang et al., 2008). Here, we present the antibiofilm activity of gurmarin, purified from *Gs* plant extract, against *Sa* under *in vitro* conditions. Further, we determined the transcriptomic analysis of *Sa* biofilm inhibition by gurmarin and confirmed its biofilm inhibitory property *in vivo* using a rat-implant biofilm model.

## Materials and methods

### Media, *Sa* biofilm growth, and screening for biofilm inhibitor

*Sa* strains NCTC 8325-4 (provided by Dr. Chia Y. Lee, University of Arkansas, AR) and clinical isolates (*S. aureus* No. 28595-1 & No. 28768-1, non MRSA, Texas Tech University Health Sciences Center, to verify biofilm inhibitory activity) were used in this study. Strain 8324-5 was used throughout in this study. Tryptic soy broth (TSB, Difco, Detroit, USA) and tryptic soy agar (TSA) with sheep blood (5%) were used as necessary. Strains were grown overnight in 10 ml of TSB at 37° C with shaking. To determine bacterial growth rate, the Bioscreen-C growth monitoring system (Oy Growth Curves Ab Ltd, Finland) was used as described before (Veerapandian and Vediyappan, 2019) with an initial inoculum of *Sa* suspension 0.09 OD_600 nm_. Overnight cultures were diluted 1:100 in fresh TSB without antibiotic and used for biofilm growth assay. A library of selectively assembled medicinal plant-derived compounds (partially purified, Laksbiotec (Pvt.), India) (Vediyappan et al., 2013) was used for initial screening purposes. Compounds were dissolved in 50% DMSO, and small aliquots (5 µl (5 µg)/100 µl TSB) were used. The criteria for isolating inhibitors of *Sa* biofilm growth were that compounds should not be toxic to *Sa* cells and should inhibit *Sa* biofilm formation. The ‘hit’ rate was about 2%. *Sa* biofilm growth with plant compounds or without (replacing solvent) was determined initially in 96-well microtiter plates (Corning, NY, USA) and secondary assay of selected fractions in glass tubes (borosilicate) under gentle shaking at 37° C.

Briefly, *Sa* suspension (10^7^/ml) in TSB was mixed with plant compounds (solubilized in 50% DMSO) and incubated for 24 h at 37 ° C. After washing off unattached cells, the adhered biofilms were measured by crystal violet (0.1%, CV) staining (Christensen et al., 1985). Experiments were repeated at least three times each with triplicates, and representative results are shown. The biofilm inhibitory proteinaceous material (gurmarin) was purified by the isoelectric focusing (IEF, preparative) method as described (Veerapandian et al., 2020) using Rotofor system (Bio-Rad, CA). The IEF purified polypeptide was pooled and dialyzed first against cold distilled water containing NaCl (0.1%) for 20 h at 4° C to remove the ampholyte and then without NaCl. The dialyzed polypeptide solution was freeze-dried, aliquoted, and stored at -80° C until further use. The purified polypeptide was evaluated for the inhibition of *Sa* biofilm at different concentrations, and the lowest concentration (1 µg/ml) that showed maximum inhibition in TSB was selected.

### SDS-PAGE and immunoblot analysis

To determine the purification of the active principle, the IEF fractions of biofilm inhibitory polypeptide were resolved on a 4-20% gradient SDS-PAGE as described (Veerapandian and Vediyappan, 2019) and stained with Coomassie brilliant blue dye solution. To determine the effect of gurmarin polypeptide on the synthesis of poly N-acetylglucosamine (PNAG) polysaccharide in *Sa* biofilm cells, cell lysates of control and peptide-treated biofilms were analyzed by immunoblot using rabbit anti-PNAG antibody (kind gift from Dr. Gerald B. Pier, Channing Laboratory, Boston) (Maira-Litran et al., 2002). Based on the protein determination, equal amounts of proteins from cell lysates of control and peptide-treated biofilms were separated on a 10% SDS-PAGE (reduced and boiled) and transferred to a PVDF membrane. The membrane was probed with an anti-PNAG primary antibody followed by an appropriate secondary antibody. Reacted polysaccharide bands were visualized by using ECL2 Western Blotting Substrate and imaging.

### Scanning electron microscope

*Sa* biofilm was grown on Thermanox plastic (Nunc, cell culture treated) without (control) or with gurmarin (1 µg/ml) using TSB in glass test tubes as mentioned above. Biofilms adhered to Thermanox plastic were fixed with 2% paraformaldehyde and 2% glutaraldehyde in PBS. The dehydrated biofilm samples were stained with uranyl acetate (2%) before viewing under an SEM (Nikon).

### Microarray protocol and analysis

Comparative DNA microarray analysis of gurmarin exposed (1 µg/ml) and unexposed (control) *Sa* cells for 16 h were conducted using *Sa* DNA microarray (version 6) from the NIH-Pathogen Functional Genomics Resource Center (PFGRC) at The Institute for Genomic Research (TIGR). The 70-mer oligo microarray was designed by PFGRC based on ORF sequences across 6 different genomes of *Sa* strains (COL, N315, Mu50, MW2, MRSA252, and MSSA476). A total of three biological replicates of the study were performed. From each biological replicate, a separate microarray analysis was performed including dye swaps. RNA preparation and array hybridization protocols recommended by PFGRC were followed. Briefly, bacterial cultures were resuspended immediately in RNAprotect Bacteria Reagent (Qiagen Inc., Valencia, CA). Total RNA of control and biofilm cells were extracted using RNeasy Protect Bacteria Mini Kit Qiagen Inc.), and DNA was removed using RNase-Free DNase set (Qiagen Inc.). RNA was quantified using spectrophotometry and the quality was confirmed by RNA gel electrophoresis. The cDNA was labeled with either CyDye3-dCTP or CyDye5-dCTP (Amersham Biosciences) using the LabelStar kit (Qiagen Inc.) and random nonamers (Sigma-Aldrich Inc., St. Louis, MO) and purified using MinElute spin column as recommended by the manufacturer. The labeled cDNA was hybridized to the microarray using Pronto Universal Hybridization Kit and the Short Oligo Hybridization solution (Corning Inc.).

Microarray hybridizations were performed by cross-hybridization of treatment and control samples. Along with the hybridizations, technical replications (dye swaps) were also performed. Images were captured using a Genepix 4000B (Molecular Devices Corporation, Union City, CA) laser scanner, and images were processed using Genepix 6.0 software (Molecular Devices). Analyses were performed using Acuity 4.0 software. Slides were normalized using standard settings (ration based so that the mean of the ratio of means, of all features, were equal to 1.0). All spots were manually evaluated, and bad, low signal, absent, or unfound features were not included. The microarrays were analyzed to obtain genes that were consistently regulated on every array with false discovery rate (FDR) (Benjamini and Hochberg, 1995). To obtain the final data provided, it was required that elements on each array and on each dye-swap (after mathematical conversion x’= -x) provided agreement and showed statistically significant similarity, based upon a student *t*-test, at a Benjamini-Hochberg false discovery rate of < 0.05 (Benjamini and Hochberg, 1995). Genes were included in the final dataset that exhibit at least 2.0 fold regulations following Lowess M log normalization.Microarray data are available in the ArrayExpress database (https://www.ebi.ac.uk/arrayexpress/experiments/E-MTAB-12154/) under accession number E-MTAB-12154.

### Bioinformatics and statistical analyses

The differentially regulated genes from the microarray study were mapped to functional classifications schemes such as Protein Information Resource (PIR) keywords, Gene Ontology terms (Ashburner et al., 2000), KEGG pathways (Ogata et al., 1999), and COG (Tatusov et al., 2003), through the use of High Throughput Gene Ontology and Functional Annotation Toolkit (HTGOFAT) (Dowd, 2005) and the database for Annotation Visualization and Integrated Discovery (DAVID) (Dennis et al., 2003). Differentially regulated genes (p<0.05) were mapped to UniProt accession numbers and Gene Index numbers using the built-in functions of HTGOFAT. These UniProt accessions were then entered into DAVID website to evaluate functional clustering and functional category enrichments.

Statistics algorithms built into Acuity 4.0 were utilized for analyses related to microarrays. Built in algorithms of the DAVID’s functional annotation tool were utilized to evaluate clustering and categorization statistics. Similarly, algorithms of the ABI Prism 7500 Sequence Detection system software (PE Applied Biosystems) were utilized for all calculations related to qRT-PCR. Correlation analyses were performed using multivariate analyses functions of JMP 5.1 (SAS Institute, Cary, NC).

### Quantitative real-time PCR

Measurements of relative transcript amounts were performed by qPCR with QuantiTect SYBR Green RT-PCR kit (Qiagen Inc.) according to the manufacturer’s instructions. Cycling conditions were based upon the standard settings recommended for the 7500 Sequence Detection System. Specific primer pairs were designed using Applied Biosystems Primer Select Software and standard settings (PE Applied Biosystems). The genes selected for final analyses using qPCR included *ddh* (forward 5-GCGTCGCTTCCCAGATATT-3; reverse 5-GATACGACCCGTACCGATAATTG-3), *lrgB* (forward 5-GCATCGTATCATCGGAGGTATT-3; reverse 5-CTGTAGTTGCTGCTTGAGGTA-3), *tenA* (forward 5-CAAAAGTTTGGCCTCCAAGTC-3; reverse 5’-CGACTATGCGCTTGGAAATACA-3), *srrA* (forward 5-TCTTTTGAAATCCATGAAGCAAGT-3; reverse 5-GCATAATTATTCTCCATTGCAAGTTC-3), and *hutG* (forward 5-TGCTATGCTTGCAGCGAAGT-3; reverse 5-GCAATATCATGTCCACCACCTAATAA-3). In addition, the 16S RNA gene (forward 5-CCGCATGGTTCAAAAGTGAAA-3; reverse 5-GCAGCGCGGATCCATCTAT-3) transcript was analyzed for both control and treatment samples, to normalize the qPCR results of selected genes. The reactions were performed on an ABI Prism 7500 Sequence Detection system (PE Applied Biosystems). The difference (fold) in the initial concentration of each transcript normalized to 16S rRNA) with respect to the control were calculated according to the comparative Ct method using the built-in functions of the 7500 system Sequence Detection Software version 1.3 (Applied Biosystems). The results of the qPCR were analyzed for correlation using multivariate analysis functions of JMP 6.0 (SAS Institute, Cary, NC).

### Rat graft (implant) biofilm model

The rat graft biofilm model mimics the implant-associated biofilms in clinical settings and is based on the published protocols (Balaban et al., 2005; Balaban et al., 2007). Wistar rats (3 weeks old, male, 200 g) were used in this study. The study protocol was approved by the Texas Tech University HSC Institutional Animal Care and Use Committee (IACUC) and conducted in an AAALAC accredited animal facility. Rats were housed in soft bed plastic cages as recommended by IACUC. Food and water were provided *ad libitum*. Three groups of rats each with 3 animals were used: (i) negative control group (grafts with no peptide, no *Sa*, but with phosphate-buffered saline (PBS) only), (ii) positive control group (grafts without peptide + *Sa*), (iii) treated animal group (grafts coated with peptide + *Sa*).

Briefly, subcutaneous pockets were made at the back of rats on one side of the median line by a 1.5 cm incision on anesthetized and shaved rats. Aseptically, 1 cm^2^ sterile Polyform graft mesh (Boston Scientific, now called eSutures, Mokena, IL) that was precoated with plasma and dried was implanted into the pocket. Immediately before implantation, the grafts were soaked without or with gurmarin (10 µg/ml) in sterile PBS for 30 minutes at room temperature. Grafts without peptide coating served as controls. The subcutaneous pockets were closed by skin clips and then 200 µl PBS only or containing exponentially growing *Sa* (2 x10^7^) were inoculated onto the graft surface. Grafts were explanted 7 days later and suspended in 1 ml of sterile PBS followed by brief sonication (10 s at low speed, Branson Ultrasonic 250). The viable *Sa* bacterial count (colony forming units, CFU) was obtained by plating the suspensions on sheep blood agar plates and incubating the plates at 37°C for 24 h. For statistical significance, two-tailed Student *t*-test available in GraphPad Prism (version 7.0) software was used. Animals were also monitored for symptoms of sickness (or death), tissue necrosis, and fluid accumulation in and around the implant-associated biofilm area of the subcutaneous pockets. In a separate animal group, the toxic effect (if any) of gurmarin peptide was tested in male Wistar rats (200 g) by oral administration of peptide (1 mg/ml in sterile PBS, one time). Rats were monitored for weight loss and symptoms of sickness or death over 7-day periods.

## Results and discussion

### Identification of *Sa* biofilm inhibitor from plant extracts and isolation

To identify inhibitors of *Sa* biofilm formation in 96-well microtiter plates, we used a focused library of medicinal plant-derived compounds and identified a compound source from one plant (*Gs*). The strategy was to select compounds that should not affect the viability or growth of the bacteria but should affect *Sa* biofilm growth as determined by crystal violet staining (Christensen et al., 1985). This plant is known to contain various small molecules with medicinal properties and a polypeptide (Imoto et al., 1991; Porchezhian and Dobriyal, 2003; Kanetkar et al., 2007). To determine whether the active principle was organic small molecules or proteinaceous, the active principle was subjected to dialysis (MWCO 1000) and proteinase-K treatment. *Sa* biofilm inhibitory principle was identified as a non-dialyzable and proteinase-K susceptible. *Gs* contains a 35-amino acid polypeptide, gurmarin, that has shown to suppress the sweet taste stimuli in rats but not in humans (Imoto et al., 1991; Kamei et al., 1992). In contrast to this activity, gymnemic acids (GAs), a family of triterpenoid small molecules, do not affect the sweet taste responses of the rat but completely suppress the sweet taste sensation in humans (Imoto et al., 1991; Kamei et al., 1992). GAs do not inhibit *Sa* biofilms under the conditions tested.

**Figure 1:**
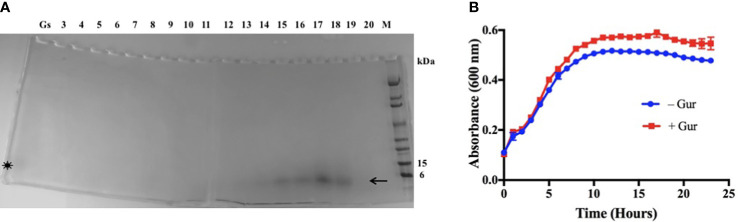
Purification of biofilm inhibitory polypeptide from *Gs* leaf extract fraction and growth of *Sa* in the presence of polypeptide. **(A)** The active principle of *Gs* leaf extract (water-ethanol, 1:1) was subjected to preparative IEF as described. Aliquots of samples from IEF fractions (3-20) were separated on SDS-PAGE (4-20%) under reduced conditions and Coomassie blue stained. Migration of the stained polypeptide (~4.2 kDa) is shown (arrow, #14-18) below to the 6 kDa protein standard. Original active fraction (Gs) was included during SDS-PAGE, and a weakly stained diffuse polypeptide band can be seen (asterisk). **(B)** Growth of *Sa* without or with purified polypeptide (15 µg/ml) in TSB at 37°C. The purified polypeptide increased slightly the growth rate of *Sa* at late exponential phase under the conditions tested.

Since the active principle was proteinaceous, we used a preparative isoelectric focusing (IEF) method to purify it (Veerapandian et al., 2020). The IEF separates proteins or polypeptides by their charge differences under electric field. Using Rotofor system with a broad-range ampholyte (pH 3-10) and the active fraction of *Gs*, the polypeptide was purified under native condition in solution (Veerapandian et al., 2020). The Coomassie blue stained IEF fractions of 4-20% gradient SDS-PAGE showed the polypeptide band with an apparent molecular weight of around 4.2 kDa in fractions #14-18 (**Figure 1A**, arrow). Imoto et al. (Imoto et al., 1991) have showed the isolation of gurmarin peptide with a similar molecular size of 4.2 kDa from *Gs* leaf extract, and our result of the gurmarin molecular size agrees with theirs. These authors isolated the polypeptide for the first time and named it ‘gurmarin’ in reference to ‘Gurmar’ which means ‘sugar destroyer’ in Hindi. We also confirmed the molecular mass of purified gurmarin by mass spectrometry (mass 4227.8, data not shown). The fractions #14-18 were pooled, dialyzed, and freeze-dried as described in the Material and methods section.

High-resolution three-dimensional solution structures of native and synthetic peptides were determined by two different research groups, and found both types of peptides were structurally and functionally similar (Arai et al., 1995; Fletcher et al., 1999). Gurmarin shows a compact structure containing antiparallel ß-hairpin (residues 22-34), several well-defined ß-turns, and a cystine-knot motif commonly observed in toxic and inhibitory polypeptides (Fletcher et al., 1999). Based on recent crystal structure and mutagenesis studies, the role of hydrophobic residues (W28, W29, and Y13 & Y14 regions) of gurmarin has been identified as a binding site for the rat sweet taste receptor, T1r2/T1r3 (Sigoillot et al., 2018). Gurmarin contains three disulfide linkages, whereby a pair of disulfides form a loop through which the third disulfide bond passes, creating a heat-stable and protease-resistant structure known as an inhibitor cystine knot (Craik et al., 2001). Knottin family of peptides is known to have diverse biological activities (Wang et al., 2008; Parthasarathy et al., 2021; Li et al., 2022). Gurmarin peptide is structurally different from the autoinducing peptide (AIP) produced by *Sa*. The active form of AIP is an eight-residue peptide with the last five residues constrained in a thiolactone ring through internal linkage to a cysteine side-chain (Ji et al., 1995). Gurmarin, on the other hand, has 6 cystines that form 3 disulfide bonds between cystines at 3-18, 10-23, and 17-33, and has two antiparallel beta strands (Arai et al., 1995; Fletcher et al., 1999).

### Gurmarin as an inhibitor of *Sa* biofilm

The purified gurmarin was verified for its effect on the growth rate of *Sa* and biofilm inhibitory activity. The polypeptide is soluble in water or growth medium. *Sa* was grown with (+ Gur) (15 µg/ml) or without (- Gur) gurmarin in microtiter wells containing TSB at 37° C in the Bioscreen-C growth monitor without shaking except for a 10-second shaking before reading absorbance at 600 nm. Results shown in **Figure 1B** indicate that *Sa* growth rate was increased slightly by gurmarin at late exponential phase. The peptide did not affect the viability of bacteria or fungi, and doesn’t hemolyze red blood cells (**Supplementary Figure 2**). *Gs* plant that produces gurmarin has been used in traditional medicines for centuries (Hooper, 1887; Kanetkar et al., 2007; Khan et al., 2019) and thus offers a safety profile for further development.

**Figure 2:**
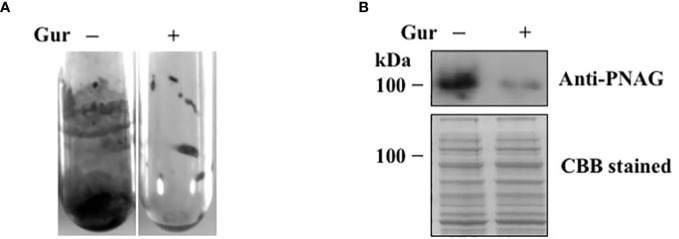
Inhibition of *Sa* biofilm growth and synthesis of polysaccharide by gurmarin polypeptide. **(A)** *Sa* biofilm growth was determined with purified gurmarin (1 µg/ml) in glass test tubes and TSB with gentle shaking (100 rpm) for 16 h at 37°C. After removing the medium and unbound cells, the biofilm was stained with crystal violet (0.1%). **(B)** Immunoblot analysis of N-acetylglucosamine polysaccharide from control and gurmarin-treated *Sa* biofilms. Control and gurmarin-treated *Sa* biofilm cell lysates with equal amounts of protein content were resolved on SDS-PAGE and immunoblotted with anti-PNAG antibody (upper panel). Biofilm proteins separated on SDS-PAGE similarly and stained with Coomassie brilliant blue (CBB) are also shown (lower panel).

We next tested the effect of gurmarin on *Sa* biofilm growth. Among different concentrations (1-10 µg/ml) of gurmarin tested, 1 µg/ml inhibited the formation of biofilm effectively (**Figure 2A**). The biofilm inhibitory concentration 50 was 0.6 µg/ml. Biofilms grown in the control tubes under gentle shaking conditions showed adhered biofilms on the test tube wall and at the bottom. *Sa* grown similarly in the presence of gurmarin (Gur +) lacks such biofilm in the test tubes suggesting gurmarin could block biofilm formation on the glass surfaces. *Sa* biofilm formation requires the production of an extracellular matrix composed of poly N-acetylglucosamine (PNAG or polysaccharide intercellular adhesin, PIA) (O'Gara, 2007). We wanted to test if gurmarin can interfere the synthesis of PNAG during *Sa* biofilm growth. Immunoblot analysis of cell lysates of control and gurmarin-treated biofilms with polyclonal anti-PNAG antibody revealed reduced synthesis of the polysaccharide in the gurmarin-treated biofilms (**Figure 2B**, upper panel). The Coomassie stained protein bands of identical samples after SDS-PAGE serve as loading controls (**Figure 2B**, lower panel). Taken together, gurmarin treatment to *Sa* reduces the synthesis of PNAG without inhibiting the growth of bacterium. PNAG is a linear ß-(1-6)-linked N-acetylglucosamine polymer synthesized by the proteins encoded by the *icaADBC* operon. In *S. aureus*, *ica*-dependent and *ica*-independent biofilm formation have been reported (O'Gara, 2007). We also tested the purified gurmarin for its inhibitory activity of biofilm growth with clinical and SH1000 strains of *Sa* and found identical inhibitory activity (**Supplementary Figure 1**, data not shown for clinical strains). While *Sa* 8324-5 is a *rsbU* negative (a 11 bp deletion in *rsbU* gene), the SH1000 strain is *rsbU* gene restored (Herbert et al., 2010), and the biofilm inhibitory activity of gurmarin is similar between these bacterial strains indicating the rsbU mutation does not affect gurmarin activity. RsbU is a member of the sigmaB operon that contains *rsbU*, *rsbV*, *rsbW*, and *sigB* genes. *rsbU* is a positive regulator, and *rsbV* and *rsbW* are the negative regulators of *sigB* in *S. aureus*. Sigma factor sigB controls hundreds of genes in response to environmental perturbations. Gurmarin did not affect preformed *Sa* biofilms.

### SEM view of gurmarin-treated *Sa* biofilm

**Figure 3:**
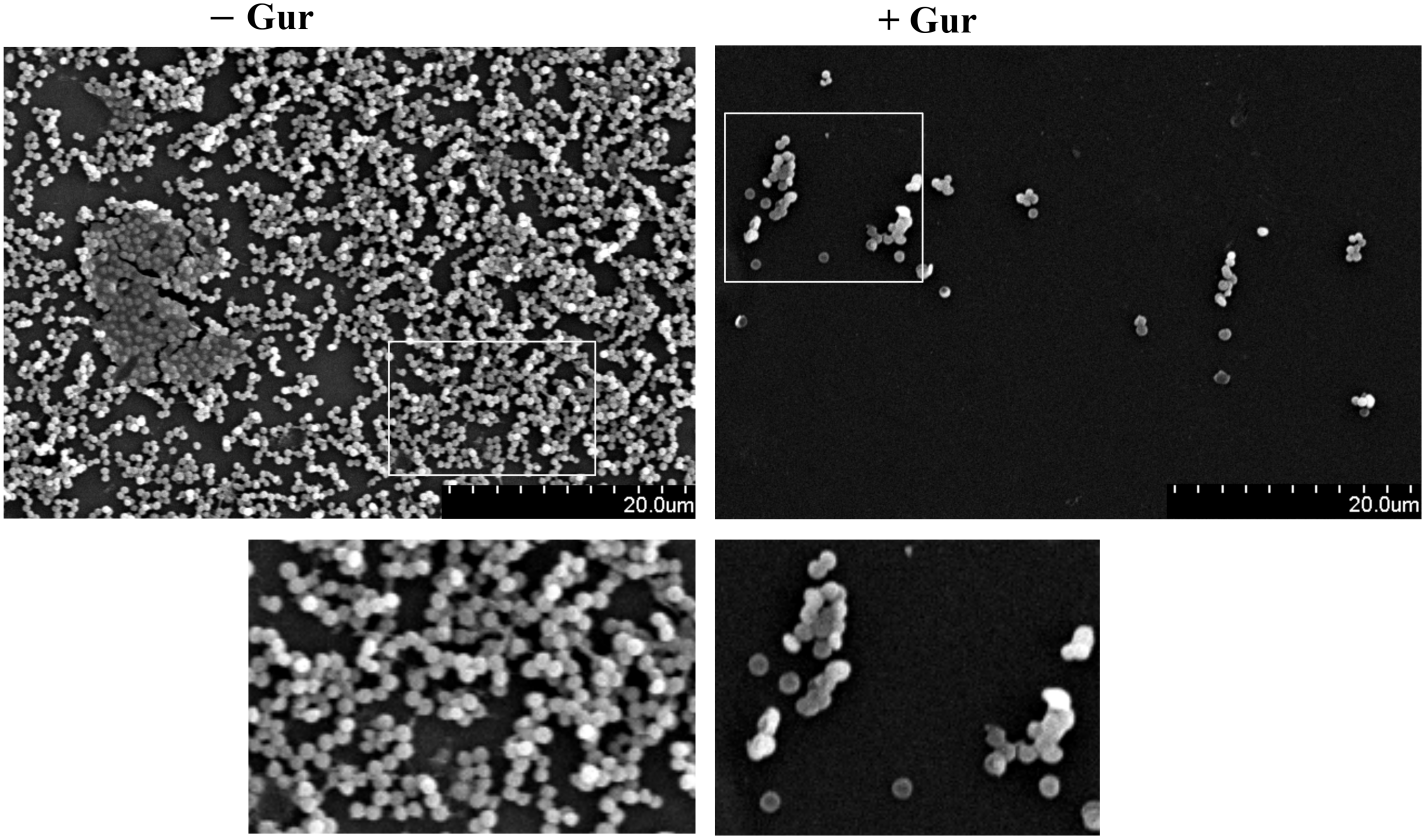
SEM view of *Sa* biofilm inhibition by gurmarin. *Sa* biofilms grown on Thermanox plastics for 16 h with gurmarin (1 µg/ml) or without were processed as described and viewed under SEM. Scale bar, 20 µm. A thick biofilm with extracellular polysaccharide fibrils is seen in the control (- Gur) and these are absent in the polypeptide-treated biofilm (+ Gur). Boxed regions are zoomed (150x).

To further evaluate if gurmarin can inhibit *Sa* biofilm formation on plastic surfaces, biofilm was developed on sterile Thermanox plastics for 16 h with gurmarin (1 µg/ml) or without in a TSB growth medium and processed for SEM. Results shown in **Figure 3** suggest that gurmarin could inhibit *Sa* biofilm formation on plastic surfaces and is in complete agreement with crystal violet stained glass biofilm assay (**Figure 2A**). It is also worth mentioning that the polysaccharide material (thick biofilms with fibrils in control, - Gur) is absent in the treated biofilm (+ Gur, SEM), which, agrees with the results of immunoblot for PNAG (**Figure 2B**).

### Gurmarin affects the expression of genes in *Sa* during its biofilm growth

**Figure 4:**
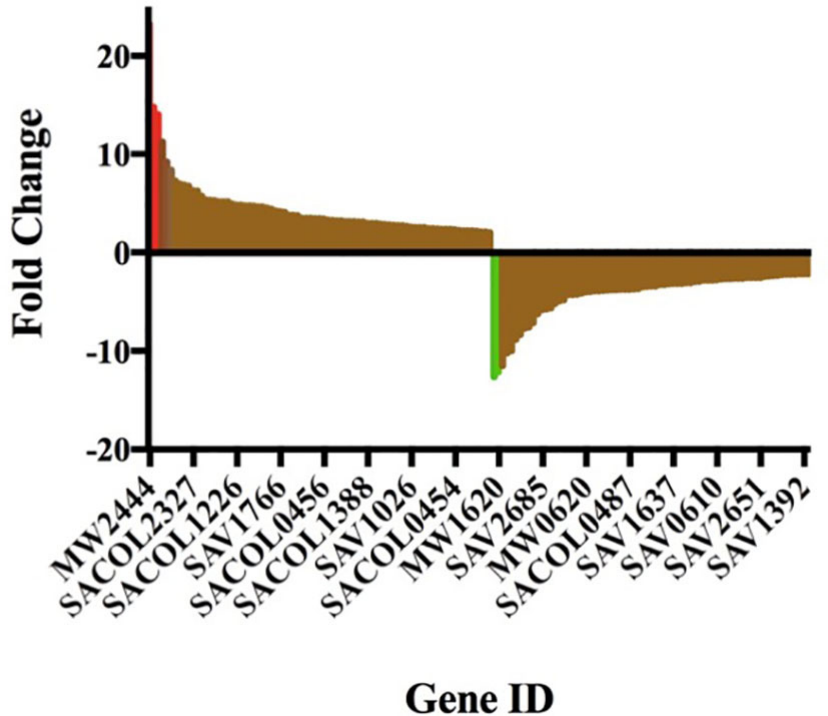
Gurmarin affects expression of *Sa* genes. Differentially expressed genes at 2-fold change is visualized in Prism 7 program. The upregulated and downregulated genes, including a few highly induced (red) and highly repressed genes (green), are shown. For gene details and fold changes, see **Tables 1** and **2**.

Gurmarin inhibits the formation of *Sa* biofilms on glass and Thermanox plastic surfaces likely by interfering with the synthesis of PNAG and other mechanisms. To understand the impact of gurmarin on *Sa* gene regulation during its biofilm growth, we performed transcriptome analyses. Comparative analysis of gurmarin exposed and unexposed (control) *Sa* cells identified a total of 161 differentially regulated genes (> 2-fold change, p < 0.05, **Figure 4**). Of these, 79 genes were upregulated (**Table 1**) and 82 genes were downregulated (**Table 2**). About 50% of the differentially regulated genes encode hypothetical proteins. The highly induced genes were *ddh* (D-2-hydroxyacid dehydrogenase (D-LDH)), *tenA* (transcriptional activator), and *eno* (enolase), etc. Gurmarin treatment to *S. aureus* may augment the catalysis of the reversible reaction of lactate to pyruvate with the reduction of NAD+ to NADH and vice versa as *ddh* was induced 23 fold (KEGG Pathway). The most repressed genes were *lrgB* (antiholin-like protein), SAV0391 GMP synthase (*guaA*), *hsdS* (restriction endonuclease subunit S), and *tagA* (teichoic acid biosynthesis protein), etc. Functional classification of the data using DAVID analysis (Dennis et al., 2003) showed clustering of upregulated genes involved in oxidoreductase activity, transferase, and metal binding (**Table 3**), while repressed functional categories included hydrolases, primary metabolism, and ATP-binding and nucleotide-binding proteins (**Table 4**). Some of the differentially expressed genes from transcriptome analysis (*e.g*. *ddh, lrgB*, and *srrA*, etc.) were confirmed by qPCR and they were generally correlated (**Table 5**).

**Table 1:** Upregulated genes in gurmarin treated *S. aureus* biofilm.

TIGR ID	Fold change	Definition	Gene symbol	FDR
MW2444	23.26	D-2-Hydroxyacid dehydrogenase	*ddh*	0.01
SACOL2086	14.77	Transcriptional regulator, TenA family	*tenA*	0.04
SACOL0842	14.04	Enolase	*eno*	0.05
SACOL2076	11.24	Conserved hypothetical protein		0.01
SAV0181	9.21	Hypothetical protein	*yagU*	0.04
SACOL2087	8.38	Hypothetical protein		0.01
MW0902	7.27	Hypothetical protein		0.01
SACOL0625	6.99	Conserved hypothetical protein		0.05
SAV1337	6.85	Guanosine 5'-monophosphage oxidoreductase		0.03
SACOL1535	6.77	DNA-binding response regulator	*srrA*	0.03
SACOL2327	6.3	Formimingoglutamase	*hutG*	0.01
MW0496	6.28	Hypothetical protein		0.01
SACOL1895	5.75	Conserved hypothetical protein		0.03
MW1484	5.38	Hypothetical protein		0.02
SACOL1759	5.32	Universal stress protein family		0.01
SAV2699	5.29	Similar to N-hydroxyarylamine O-acetyltransferase		0.03
SAV2479	5.19	Hypothetical protein		0.05
SACOL0522	5.19	Recombinant protein Rec R	*recR*	0.04
SAV0382	5.17	Similar to nitro/flavin reductase		0.05
SACOL1767	4.94	Conserved hypothetical protein	*ezrA*	0.02
SACOL1226	4.86	Conserved hypothetical protein		0.03
MW1039	4.85	Hypothetical protein		0.02
SAV1765	4.76	Lysophospholipase homolog		0.04
SAV1761	4.75	Multidrug resistance protein homolog		0.02
SACOL2119	4.74	CTP synthase	*pyrG*	0.03
SAV2029	4.67	GroEL protein	*groEL*	0.03
SAV2305	4.64	Glycerate dehydrogenase		0.03
SACOL1245	4.54	3-oxoacyl-(acyl-carrier-protein) reductase	*fabG1*	0.04
SACOL1437	4.42	Cold shock protein, CSD family	*cspA*	0.02
MW2014	4.23	Thiamin phosphate synthase (chain B)	*thiE*	0.03
SAV1766	4.17	Proline dehydrogenase homolog		0.02
SAV0004	4.11	Recombinant protein F	*recF*	0.02
SACOL1953	3.81	Hypothetical protein		0.01
MA1210	3.8	Hypothetical protein		0.05
SACOL0490	3.74	Hypothetical protein		0.01
SAV2442	3.51	Similar to dTDP-glucose 4,6-dehydratase		0.05
MW0363	3.51	Hypothetical protein		0.03
SACOL1372	3.5	Hypothetical protein		0.02
SACOL2304	3.47	Conserved domain protein		0.01
SACOL1068	3.44	Quinol oxidase, subunit III	*qoxC*	0.05
SACOL0456	3.4	Conserved hypothetical protein		0.01
SAV2631	3.3	Transcriptional regulator		0.05
MW1830	3.28	Hypothetical protein		0.04
SACOL2082	3.23	Membrane protein, putative		0.02
SACOL2241	3.23	Conserved hypothetical protein		0.01
SAV0169	3.16	Hypothetical protein		0.05
MW2072	3.15	Hypothetical protein		0.02
SAV1578	3.14	Probable methyltransferase		0.02
SAV1080	3.1	Hypothetical protein		0.04
SACOL1321	3.1	Aerobic glycerol-3-phosphate dehydrogenase	*glpD*	0.05
SACOL1388	2.96	Conserved hypothetical protein		0.02
SAV2328	2.95	Dehydrogenase		0.02
SACOL2138	2.95	Cation efflux family protein	*czrB*	0.03
SACOL0541	2.9	spoVG protein	*spoVG*	0.01
SAV0240	2.85	Putative flavohemoprotein		0.04
SAV2186	2.83	Similar to alginate lyase		0.02
SAV1241	2.78	50S ribosomal protein L19	*rplS*	0.05
SACOL0210	2.76	Hypothetical protein		0.03
SACOL1176	2.74	Hypothetical protein		0.02
SACOL1693	2.66	Preprotein translocase, YajC subunit	*yajC*	0.03
SAV1026	2.59	Competence transcription factor	*comK*	0.04
SAV0508	2.56	Hypothetical protein		0.02
SAV2263	2.54	Hypothetical protein		0.02
SAV0420	2.54	Hypothetical protein		0.02
SACOL0878	2.45	Hypothetical protein		0.02
MW2112	2.43	Hypothetical protein		0.01
SACOL2156	2.39	ATP-binding protein, Mrp-Nbp35 family		0.01
SAV1275	2.37	Similar to metallo-beta-lactamase family protein		0.04
SACOL1064	2.36	Conserved hypothetical protein		0.02
SAV1535	2.35	Glycine dehydrogenase subunit 2	*gcvP*	0.03
SACOL0454	2.34	Sodium dicarboxylate symporter family protein		0.03
SAV2129	2.26	Similar to spermine/spermidine acetyltransferase		0.04
SAV1222	2.23	ribuloase-5-phosphate 3-epimerase homolog	*cfxE*	0.02
MW0477	2.23	Hypothetical protein	*ctsR*	0.02
SAV1480	2.21	Thioredoxin reductase-like protein	*trxB*	0.02
SAV2317	2.17	Hypothetical protein		0.02
SAV2351	2.1	Hypothetical protein		0.01
SAV1747	2.09	Similar to metal-dependent hydrolase		0.03
SACOL0124	2.02	Phosphopentomutase	*deoB*	0.01

**Table 2:** Down regulated genes in gurmarin treated *S. aureus* biofilm.

TIGR ID	Fold change	Definition	Gene symbol	FDR
MW0239	-12.64	Antiholin-like protein	*lrgB*	0.03
MW1620	-12.13	Hypothetical protein		0.05
SAV0391	-11.49	GMP synthase (guaA) glutamine hydrolyzing	*guaA*	0.02
SAV0432	-10.24	Probable restriction modification system specificity subunit	*hsdS*	0.01
SAV1618	-9.99	Alanyl-tRNA synthetase	*alaS*	0.01
SAV0195	-8.85	Probable type I restriction enzyme restriction chain	*hsdR*	0.02
SACOL0712	-8.35	Lipase-esterase		0.02
SACOL0852	-7.72	Hypothetical protein		0.02
SAV2169	-7.62	Probable multidrug transporter		0.02
SAV0463	-7.16	ABC transporter permease protein		0.05
MW0416	-6.24	Hypothetical protein		0.01
SAV2685	-5.84	Similar to integral membrane protein		0.01
SAV2323	-5.76	PTS system, arbutin-like IIBC component	*glvC*	0.04
SAV1345	-5.65	Similar to exonuclease	*sbcD*	0.01
SACOL2056	-5.21	Anti-anti-sigma factor	*rsbV*	0.05
SAV1217	-4.95	Similar to RNA-binding Sun protein		0.02
SAV0636	-4.83	Teichoic acid biosynthesis protein	*tagA*	0.02
SAV1592	-4.31	Similar to iojap protein family		0.01
SACOL0598	-4.3	L-ribulokinase. Putative		0.02
SAV0179	-4.26	Similar to surfactin synthase		0.05
SACOL1738	-4.16	Hypothetical protein		0.05
MW0620	-4.04	Hypothetical protein		0.01
SAV0464	-3.99	Lactococcal lipoprotein		0.03
SAV2052	-3.94	Similar to ATP/GTP hydrolase		0.02
SAV2057	-3.92	2-isopropylmalate synthase	*leuA*	0.02
SACOL1533	-3.9	Lipoprotein, putative		0.03
SAV1879	-3.86	Aminopeptidase	*ampS*	0.03
SACOL1649	-3.81	Conserved hypothetical protein		0.02
SAV2445	-3.81	Hypothetical protein		0.04
SAV0839	-3.8	Similar to ABC transporter substrate-binding protein		0.01
SAV1912	-3.79	NAD(+) synthase	*nadE*	0.02
SACOL0487	-3.78	hypothetical protein		0.03
MW1645	-3.73	Hypothetical protein		0.03
SACOL2710	-3.71	Conserved hypothetical protein		0.02
SAV1762	-3.52	Similar to Fe-S oxidoreductase		0.01
SACOL1568	-3.48	Exodeoxyribonuclease VII, large subunit	*xseA*	0.03
SACOL0716	-3.45	DNA-binding response regulator		0.01
SACOL1530	-3.45	Lipoprotein, putative		0.03
SAV0327	-3.3	Putative lipoate-protein ligase		0.01
SACOL2496	-3.28	Conserved hypothetical protein		0.02
SAV0236	-3.21	Hypothetical protein		0.03
SAV1637	-3.2	Protein-export membrane protein	*secF*	0.01
MW0459	-3.18	Hypothetical protein		0.02
SAV1913	-3.17	Nicotinate phosphoribosultransferase		0.01
SACOL0549	-3.11	Tetrapyrrole methylase family protein		0.04
SACOL0297	-3.1	Conserved hypothetical protein		0.04
MW0509	-3.01	Branched-chain amino acid aminotransferase	*ilvE*	0.01
SACOL1230	-2.97	Conserved hypothetical protein		0.03
SAV1896	-2.85	Hypothetical protein		0.05
SAV0756	-2.85	Probable HD superfamily hydrolase		0.04
MW1853	-2.83	NAD(+) synthase	*nadE*	0.01
SAV0610	-2.79	Similar to iron(III) ABC transporter permease protein		0.04
SAV2456	-2.75	Hypothetical protein		0.03
SAV0314	-2.68	Similar to putative sodium/glucose cotransporter		0.01
SACOL0663	-2.68	Arginyl-tRNA synthetase	*argS*	0.03
SAV0718	-2.67	Hypothetical protein		0.02
SAV1482	-2.67	Probable ATP-dependent DNA helicase	*recQ*	0.03
SACOL1518	-2.66	Cytidylate kinase	*cmk*	0.01
SACOL2054	-2.59	RNA polymerase sigma-37 factor	*rpoF*	0.02
SAV1737	-2.59	3-deoxy-7-phosphoheptulonate synthase		0.02
SACOL2353	-2.58	Transcriptional regulator	*tcaR*	0.05
SAV2651	-2.57	Hypothetical protein		0.04
MW2255	-2.46	Hypothetical protein		0.02
SACOL0962	-2.45	Glycerophosphoryl diester phosphodiesterase GlpQ, putative	*glpQ*	0.04
SACOL1410	-2.39	FemA protein	*femA*	0.01
SACOL1997	-2.35	Transcriptional regulator, GntR family		0.04
SACOL0485	-2.28	Staphylococcus tandem lipoprotein		0.02
SACOL0652	-2.25	Conserved hypothetical protein		0.03
SAV0564	-2.25	Similar to poly (glycerol-phosphate) alpha-glucosyltransferase	0.03
SACOL2548	-2.24	Conserved hypothetical protein		0.02
SAV1598	-2.21	Putative lipase		0.05
SAV1392	-2.2	ABC transporter homolog		0.03
MW1750	-2.2	Probable specificity determinant	*hsdS*	0.02
SACOL2255	-2.18	Conserved hypothetical protein		0.04
SAV2031	-2.15	Hypothetical protein		0.03
SAV1596	-2.12	Shikimate dehydrogenase	*aroE*	0.02
SAV0363	-2.12	GTP-binding protein		0.02
SACOL1793	-2.11	Conserved hypothetical protein	*ytpQ*	0.01
SAV1183	-2.08	UDP-N-acetylmuramoyl-L-alanyl-D-glutamate synthase	*murD*	0.02
MW2067	-2.06	Hypothetical protein		0.04
SACOL1390	-2.05	DNA topoisomerase IV, A subunit	*parC*	0.02

**Table 3:** Induced functional categories.

Category	Term	Count	%	P-Value
Swiss-Prot PIR Keywords	Oxidoreductase	10	12.7%	9.90E-04
Gene Ontology Molecular Function	Transferase	7	8.9%	7.60E-03
Swiss-Prot PIR Keywords	Cell Cycle	3	3.8%	1.50E-02
Swiss-Prot PIR Keywords	Cellular metabolism	5	6.3%	2.40E-02
Swiss-Prot PIR Keywords	Membrane protein	5	6.3%	2.70E-02
Swiss-Prot PIR Keywords	Metal-binding	7	8.9%	2.90E-02

**Table 4:** Repressed functional categories.

Category	Term	Count	%	P-Value
Swiss-Prot PIR Keywords	Transferase	9	10.9%	2.80E-03
Swiss-Prot PIR Keywords	Hydrolase	11	13.4%	6.80E-03
Swiss-Prot PIR Keywords	Metal-binding	8	9.8%	7.00E-03
Swiss-Prot PIR Keywords	Nucleotide-binding	9	10.9%	8.70E-03
Swiss-Prot PIR Keywords	ABC protein	4	4.9%	2.70E-02
Gene Ontology Molecular Functions	Ligase	6	7.3%	3.30E-02
Swiss-Prot PIR Keywords	Amino-acid biosynthesis	5	6.1%	3.30E-02
Gene Ontology Molecular Functions	Primary metabolism	27	32.9%	4.00E-02

**Table 5:** Correlation of microarray data with quantitative Real-time PCR (qPCR, fold change).

Gene	Microarray	qPCR
*ddh*	23.2	18
*hutG*	6.3	2.0
*lrgB*	-12.6	-6.2
*srrA*	6.8	4.0
*tenA*	14.8	4.2

Oxidoreductase. These are one of the upregulated groups of genes in *Sa* biofilms by gurmarin. The generation of oxidative or nitrosative stress has been reported to affect extracellular matrix and biofilm formation in *Sa* (Schlag et al., 2007; Arce Miranda et al., 2011). Ten oxidoreductase genes (**Table 6**), including dehydrogenase and reductase genes, were found highly induced in gurmarin-treated *Sa* biofilm suggesting gurmarin may be causing oxidative or nitrosative stress, leading to the expression of oxidoreductase genes. One nitric oxide reductase gene was also found upregulated (**Table 1**, SAV0382, 5.17 fold). Lewis et al. (Lewis et al., 2015) have shown that this gene (*nor*) was upregulated during low-oxygen growth or in a cell population near the biofilm substratum and dependent on SrrAB, a two-component system that regulates the expression of respiration and nitrosative stress resistance genes. The *srrA* gene (response regulator) was found upregulated in peptide exposed *Sa* biofilm (**Table 1**, SACOL1535, 6.7 fold).

**Table 6:** Genes identified in the oxidoreductase category (induced).

Gene ID (TIGR)	Fold change	Definition	Gene symbol	FDR
MW2444	23.26	D-2-hydroxyacid dehydrogenase	*ddh*	0.01
SAV1337	6.85	Guanosine 5’-monophosphate oxidoreductase		0.03
SAV0382	5.17	Similar to nitro/flavin reductase		0.05
SACOL1245	4.54	3-oxoacyl-(acyl-carrier-protein) reductase	*fabG1*	0.04
SACOL1068	3.44	Quinol oxidase, subunit III	*qoxC*	0.05
SACOL1321	3.10	Aerobic glycerol-3-phosphate dehydrogenase	*glpD*	0.05
SAV2328	2.95	Dehydrogenase		0.02
SAV2186	2.83	Similar to alginate lyase		0.02
MW2112	2.43	Hypothetical protein		0.01
SAV1535	2.35	Glycine dehydrogenase subunit 2	*gcvP*	0.03

**Hydrolases** (downregulated category). Eleven hydrolase genes were downregulated 2-8 fold, including lipase-esterase, exonuclease, and aminopeptidase (**Table 7**). *lrgB* gene is one of the highly repressed genes (**Table 2**, MW0230,-12.6 fold). The *lrg* and *cid* operons work inversely. The *cid* operon (*cidA, cidB*) affects murein hydrolase activity positively as disruption of the *cidA* gene reduces extracellular murein hydrolase activity (Rice et al., 2003) (holin) and the *lrg* operon (*lrgA, lrgB*) impacts murein hydrolase activity negatively (antiholin) as a *lrgAB* mutation increases murein hydrolase activity (Groicher et al., 2000; Rice et al., 2003; Rice et al., 2005; Bayles, 2007). Based on our data (inhibition of biofilm, downregulation of *lrgB* & *lrgA*), gurmarin exposure to *Sa* may increase murein hydrolases targeting their substrate, the cell wall peptidoglycan (PG), resulting defect in adhered biofilm and or enhanced dispersal of cells.

**Table 7:** Genes identified in the hydrolase category (repressed).

Gene ID (TIGR)	Fold change	Definition	Gene symbol	FDR
SACOL0712	-8.35	Lipase-esterase		0.02
SAV1345	-5.65	Exonuclease, hydrolase		0.01
SACOL1738	-4.16	Hypothetical protein		0.05
SAV1879	-3.86	Aminopeptidase	*ampS*	0.03
SACOL1649	-3.81	Hydrolase		0.02
SACOL1568	-3.48	Exonuclease, nuclease	*xseA*	0.03
SAV0756	-2.85	Hydrolase		0.04
SAV1482	-2.67	ATP-binding, hydrolase		0.03
SAV2651	-2.57	Hydrolase		0.04
SACOL0962	-2.45	Hydrolase		0.04
SAV2031	-2.15	protease		0.03

In addition to peptidoglycan, *Sa* contains capsular polysaccharide (CP), cell wall teichoic acid (WTA), and PNAG/PIA as major surface polysaccharide components of the cell envelop, and each play distinct roles in *Sa* biofilm formation and pathogenesis. TagA is one of the early enzymes involved in the synthesis of *t*eichoic *a*cid *g*lycerol (*tag*) by catalyzing the transfer of an N-acetylmannosamine residue to a lipid (Sewell and Brown, 2014). Gurmarin downregulated the expression of *tagA* (**Table 2**, SAV0636, -4.83 fold) indicating the inhibitory effect of gurmarin on the synthesis of cell wall components and PNAG polysaccharides (**Figure 2**). Studies have shown that increased wall teichoic acid production and D-alanylation contribute to daptomycin antibiotic-resistance phenotypes in clinical isolates of MRSA (Bertsche et al., 2011; Bertsche et al., 2013).

### Single exposure of gurmarin to *Sa* reduces its biofilm formation and virulence in a Rat-graft biofilm model

**Figure 5:**
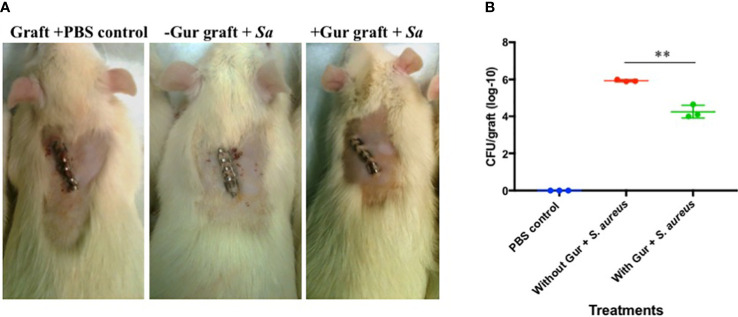
Rat-graft model of biofilm assay. **(A)** A representative animal for each group 7 days post-infection is shown. Small graft mesh (1 cm^2^) soaked in PBS (control) or gurmarin solubilized in PBS (treated) was placed in a subcutaneous pocket on the median shoulder of an anesthetized rat and closed with skin clips. Immediately, PBS (negative control group) or *Sa* suspension in PBS (positive control group and treatment group) were instilled in the respective group of animals at the closures and monitored for 7 days. Note, the middle image has ‘subcutaneous swelling’ and ‘subdermal discoloration’ both consistent with necrocellulitis. **(B)** Seven days after implantation, grafts were retrieved from the pockets and determined for the *Sa* biofilm cells (CFU). ** *p* < 0.002.

Since gurmarin inhibited *Sa* biofilm growth *in vitro* and affected its gene expression on a global scale, we evaluated the biofilm inhibitory activity of gurmarin using a rat-graft biofilm model (Balaban et al., 2005; Balaban et al., 2007) with small groups of animals. Grafts without and with peptide coating (one time) were implanted in the tissue pockets and immediately inoculated with *Sa* suspension. The development of *Sa* biofilms on the implants was determined seven days-post infection by CFU counts. While all three groups of the animals were normal during the 7-days (**Figure 5A**), the tissue around the implants of the positive control (grafts without gurmarin + *Sa*) showed necrosis and fluid accumulation, after opening the pockets. These symptoms were absent in the negative control (grafts + PBS) and the treated (gurmarin-coated grafts + *Sa*) groups of animals. The CFU determination showed a two-log reduction of *Sa* in peptide-treated implants when compared to the peptide-uncoated implants (**Figure 5B**). These *in vivo* results agree with the *in vitro* results where gurmarin inhibited the formation of *Sa* biofilm on abiotic surfaces. To determine if gurmarin causes any toxicity to animals, we administered the polypeptide solution (1 mg/ml/rat, once) to rats orally and monitored for them 7 days. The polypeptide did not cause any death or visible side effects to animals. This result agrees with the nontoxic effect of gurmarin on *Sa* (**Figure 1B**), yeast cells, and red blood cells (**Supplementary Figure 2**). Further studies are required to evaluate the efficacy of gurmarin using additional *Sa* (*e.g*. clinical) strains and animal infection models with larger groups.

## Conclusion

The gurmarin polypeptide isolated from *Gs* plant extract showed inhibitory activity against *Sa* biofilm growth both *in vitro* and *in vivo* without affecting the bacterial viability. SEM and biochemical analyses for PNAG correlate with the results of biofilm inhibition. Further, exposure of gurmarin to *Sa* affected its gene expression on a global scale, including regulatory genes involved in peptidoglycan, cell envelope synthesis, and other functions contributing the biofilm inhibition. Further work on identifying the cellular target(s) of gurmarin in *Sa* could reveal its mechanism of action.

## Data availability statement

The data presented in the study are deposited in the ArrayExpress repository (https://www.ebi.ac.uk/arrayexpress/experiments/E-MTAB-12154/), accession number E-MTAB-12154.

## Ethics statement

The animal study was reviewed and approved by Texas Tech University Health Sciences Center Institutional Animal Care and Use Committee (IACUC), Lubbock, TX, United States.

## Author contributions

Conceptualization: GV, SD, JF, and GB; methodology and data analyses: GV, AC, SD, and GB; writing, review, editing: GV, AC, SD, GB, and JF. All authors contributed to the article and approved the submitted version.

## Funding

We thank Texas Tech University for an initial seed grant to GV and JF. Funding sources from the Division of Biology and Johnson Cancer Research Center (JCRC) as BRIEF and IRA awards, respectively, to GV, are kindly acknowledged. We also thank the K-INBRE Core Facility support to GV. We are grateful to JCRC, and the College of Arts and Sciences, KSU for undergraduate scholarships to AC. The publication of this article was financed, in part, with support from the Kansas State University Open Access Publishing Fund.

## Acknowledgments

We thank Pathogen Functional Genomics Resource Center (PFGRC) and The Institute for Genomic Research (TIGR) for supplying *Sa* DNA microarray. We are grateful to Gerald B. Pier for providing anti-PNAG antibody generously. We also thank Chia Y. Lee and Alexander R. Horswill for *Sa* strains and plasmids.

## Conflict of interest

The authors declare that the research was conducted in the absence of any commercial or financial relationships that could be construed as a potential conflict of interest.

## Publisher’s note

All claims expressed in this article are solely those of the authors and do not necessarily represent those of their affiliated organizations, or those of the publisher, the editors and the reviewers. Any product that may be evaluated in this article, or claim that may be made by its manufacturer, is not guaranteed or endorsed by the publisher.

## References

[B1] Arai K. Ishima R. Morikawa S. Miyasaka A. Imoto T. Yoshimura S. . (1995). Three-dimensional structure of gurmarin, a sweet taste-suppressing polypeptide. J. Biomol. NMR 5 (3), 297–305. doi: 10.1007/BF00211756 7787425

[B2] Arce Miranda J. E. Sotomayor C. E. Albesa I. Paraje M. G. (2011). Oxidative and nitrosative stress in *Staphylococcus aureus* biofilm. FEMS Microbiol. Lett. 315 (1), 23–29. doi: 10.1111/j.1574-6968.2010.02164.x 21134223

[B3] Ashburner M. Ball C. A. Blake J. A. Botstein D. Butler H. Cherry J. M. . (2000). Gene ontology: tool for the unification of biology. the gene ontology consortium. Nat. Genet. 25 (1), 25–29. doi: 10.1038/75556 10802651 PMC3037419

[B4] Balaban N. Cirioni O. Giacometti A. Ghiselli R. Braunstein J. B. Silvestri C. . (2007). Treatment of *Staphylococcus aureus* biofilm infection by the quorum-sensing inhibitor RIP. Antimicrob. Agents Chemother. 51 (6), 2226–2229. doi: 10.1128/AAC.01097-06 17371825 PMC1891383

[B5] Balaban N. Stoodley P. Fux C. A. Wilson S. Costerton J. W. Dell'Acqua G. (2005). Prevention of staphylococcal biofilm-associated infections by the quorum sensing inhibitor RIP. Clin. Orthop. Relat. Res. 437), 48–54. doi: 10.1097/01.blo.0000175889.82865.67 16056025

[B6] Bayles K. W. (2007). The biological role of death and lysis in biofilm development. Nat. Rev. Microbiol. 5 (9), 721–726. doi: 10.1038/nrmicro1743 17694072

[B7] Benjamini Y. Hochberg Y. (1995). Controlling the false discovery rate: A practical and powerful approach to multiple testing. J. R. Stat. Soc. Ser. B 57, 289–300. doi: 10.1111/j.2517-6161.1995.tb02031.x

[B8] Bertsche U. Weidenmaier C. Kuehner D. Yang S. J. Baur S. Wanner S. . (2011). Correlation of daptomycin resistance in a clinical *Staphylococcus aureus* strain with increased cell wall teichoic acid production and d-alanylation. Antimicrob. Agents Chemother. 55 (8), 3922–3928. doi: 10.1128/AAC.01226-10 21606222 PMC3147621

[B9] Bertsche U. Yang S. J. Kuehner D. Wanner S. Mishra N. N. Roth T. . (2013). Increased cell wall teichoic acid production and d-alanylation are common phenotypes among daptomycin-resistant methicillin-resistant *Staphylococcus aureus* (MRSA) clinical isolates. PloS One 8 (6), e67398. doi: 10.1371/journal.pone.0067398 23785522 PMC3681945

[B10] Brady R. A. Leid J. G. Calhoun J. H. Costerton J. W. Shirtliff M. E. (2008). Osteomyelitis and the role of biofilms in chronic infection. FEMS Immunol. Med. Microbiol. 52 (1), 13–22. doi: 10.1111/j.1574-695X.2007.00357.x 18081847

[B11] Cassat J. E. Lee C. Y. Smeltzer M. S. (2007). Investigation of biofilm formation in clinical isolates of *Staphylococcus aureus* . Methods Mol. Biol. 391, 127–144. doi: 10.1007/978-1-59745-468-1_10 18025674 PMC4098860

[B12] Cegelski L. Marshall G. R. Eldridge G. R. Hultgren S. J. (2008). The biology and future prospects of antivirulence therapies. Nat. Rev. Microbiol. 6 (1), 17–27. doi: 10.1038/nrmicro1818 18079741 PMC2211378

[B13] Christensen G. D. Simpson W. A. Younger J. J. Baddour L. M. Barrett F. F. Melton D. M. . (1985). Adherence of coagulase-negative staphylococci to plastic tissue culture plates: a quantitative model for the adherence of staphylococci to medical devices. J. Clin. Microbiol. 22 (6), 996–1006. doi: 10.1128/jcm.22.6.996-1006.1985 3905855 PMC271866

[B14] Colgrave M. L. Craik D. J. (2004). Thermal, chemical, and enzymatic stability of the cyclotide kalata B1: the importance of the cyclic cystine knot. Biochemistry 43 (20), 5965–5975. doi: 10.1021/bi049711q 15147180

[B15] Craik D. J. Daly N. L. Waine C. (2001). The cystine knot motif in toxins and implications for drug design. Toxicon 39 (1), 43–60. doi: 10.1016/s0041-0101(00)00160-4 10936622

[B16] Davies D. (2003). Understanding biofilm resistance to antibacterial agents. Nat. Rev. Drug Discovery 2 (2), 114–122. doi: 10.1038/nrd1008 12563302

[B17] del Pozo J. L. Patel R. (2007). The challenge of treating biofilm-associated bacterial infections. Clin. Pharmacol. Ther. 82 (2), 204–209. doi: 10.1038/sj.clpt.6100247 17538551

[B18] DennisG.Jr. ShermanB. T. HosackD. A. YangJ. GaoW. LaneH. C. . (2003). DAVID: Database for annotation, visualization, and integrated discovery. Genome Biol. 4 (5), P3. doi: 10.1186/gb-2003-4-9-r60 12734009

[B19] Donlan R. M. Costerton J. W. (2002). Biofilms: survival mechanisms of clinically relevant microorganisms. Clin. Microbiol. Rev. 15 (2), 167–193. doi: 10.1128/CMR.15.2.167-193.2002 11932229 PMC118068

[B20] Dowd S. E. (2005). HTGOFAT: High throughput gene ontology and functional annotation toolkit. Available at: https://www.mrdnalab.com [Accessed August 12, 2022]

[B21] Dowd S. E. Wolcott R. D. Sun Y. McKeehan T. Smith E. Rhoads D. (2008). Polymicrobial nature of chronic diabetic foot ulcer biofilm infections determined using bacterial tag encoded FLX amplicon pyrosequencing (bTEFAP). PloS One 3 (10), e3326. doi: 10.1371/journal.pone.0003326 18833331 PMC2556099

[B22] Flemming H. C. Wingender J. (2010). The biofilm matrix. Nat. Rev. Microbiol. 8 (9), 623–633. doi: 10.1038/nrmicro2415 20676145

[B23] Fletcher J. I. Dingley A. J. Smith R. Connor M. Christie M. J. King G. F. (1999). High-resolution solution structure of gurmarin, a sweet-taste-suppressing plant polypeptide. Eur. J. Biochem. 264 (2), 525–533. doi: 10.1046/j.1432-1327.1999.00659.x 10491100

[B24] Gotz F. (2002). Staphylococcus and biofilms. Mol. Microbiol. 43 (6), 1367–1378. doi: 10.1046/j.1365-2958.2002.02827.x 11952892

[B25] Groicher K. H. Firek B. A. Fujimoto D. F. Bayles K. W. (2000). The *Staphylococcus aureus* lrgAB operon modulates murein hydrolase activity and penicillin tolerance. J. Bacteriol. 182 (7), 1794–1801. doi: 10.1128/JB.182.7.1794-1801.2000 10714982 PMC101860

[B26] Harraghy N. Seiler S. Jacobs K. Hannig M. Menger M. D. Herrmann M. (2006). Advances in *in vitro* and *in vivo* models for studying the staphylococcal factors involved in implant infections. Int. J. Artif. Organs 29 (4), 368–378. doi: 10.1177/039139880602900406 16705605

[B27] Harriott M. M. Noverr M. C. (2009). *Candida albicans* and *Staphylococcus aureus* form polymicrobial biofilms: effects on antimicrobial resistance. Antimicrob. Agents Chemother. 53 (9), 3914–3922. doi: 10.1128/AAC.00657-09 19564370 PMC2737866

[B28] Herbert S. Ziebandt A. K. Ohlsen K. Schafer T. Hecker M. Albrecht D. . (2010). Repair of global regulators in *Staphylococcus aureus* 8325 and comparative analysis with other clinical isolates. Infect. Immun. 78 (6), 2877–2889. doi: 10.1128/IAI.00088-10 20212089 PMC2876537

[B29] Hooper D. (1887). An examination of the leaves of *Gymnema sylvestre* . Nat. London 35, 565–567. doi: 10.1038/035565a0

[B30] Imoto T. Miyasaka A. Ishima R. Akasaka K. (1991). A novel peptide isolated from the leaves of *Gymnema sylvestre*–i. characterization and its suppressive effect on the neural responses to sweet taste stimuli in the rat. Comp. Biochem. Physiol. A Comp. Physiol. 100 (2), 309–314. doi: 10.1016/0300-9629(91)90475-r 1685952

[B31] Ji G. Beavis R. C. Novick R. P. (1995). Cell density control of staphylococcal virulence mediated by an octapeptide pheromone. Proc. Natl. Acad. Sci. U.S.A. 92 (26), 12055–12059. doi: 10.1073/pnas.92.26.12055 8618843 PMC40295

[B32] Kamei K. Takano R. Miyasaka A. Imoto T. Hara S. (1992). Amino acid sequence of sweet-taste-suppressing peptide (gurmarin) from the leaves of *Gymnema sylvestre* . J. Biochem. 111 (1), 109–112. doi: 10.1093/oxfordjournals.jbchem.a123705 1607357

[B33] Kanetkar P. Singhal R. Kamat M. (2007). *Gymnema sylvestre*: A memoir. J. Clin. Biochem. Nutr. 41 (2), 77–81. doi: 10.3164/jcbn.2007010 18193099 PMC2170951

[B34] Kavanaugh J. S. Horswill A. R. (2016). Impact of environmental cues on staphylococcal quorum sensing and biofilm development. J. Biol. Chem. 291 (24), 12556–12564. doi: 10.1074/jbc.R116.722710 27129223 PMC4933443

[B35] Khan F. Sarker M. M. R. Ming L. C. Mohamed I. N. Zhao C. Sheikh B. Y. . (2019). Comprehensive review on phytochemicals, pharmacological and clinical potentials of *Gymnema sylvestre* . Front. Pharmacol. 10 1223. doi: 10.3389/fphar.2019.01223 31736747 PMC6830388

[B36] Leach M. J. (2007). *Gymnema sylvestre* for diabetes mellitus: a systematic review. J. Altern. Complement Med. 13 (9), 977–983. doi: 10.1089/acm.2006.6387 18047444

[B37] Lewis A. M. Matzdorf S. S. Endres J. L. Windham I. H. Bayles K. W. Rice K. C. (2015). Examination of the *Staphylococcus aureus* nitric oxide reductase (saNOR) reveals its contribution to modulating intracellular NO levels and cellular respiration. Mol. Microbiol. 96 (3), 651–669. doi: 10.1111/mmi.12962 25651868 PMC4554693

[B38] Li C. Y. Rehm F. B. H. Yap K. Zdenek C. N. Harding M. D. Fry B. G. . (2022). Cystine knot peptides with tuneable activity and mechanism. Angew Chem. Int. Ed Engl. 61 (19), e202200951. doi: 10.1002/anie.202200951 35224831 PMC9539897

[B39] Mah T. F. Pitts B. Pellock B. Walker G. C. Stewart P. S. O'Toole G. A. (2003). A genetic basis for *Pseudomonas aeruginosa* biofilm antibiotic resistance. Nature 426 (6964), 306–310. doi: 10.1038/nature02122 14628055

[B40] Maira-Litran T. Kropec A. Abeygunawardana C. Joyce J. Mark G. Goldmann D. A. . (2002). Immunochemical properties of the staphylococcal poly-n-acetylglucosamine surface polysaccharide. Infect. Immun. 70 (8), 4433–4440. doi: 10.1128/IAI.70.8.4433-4440.2002 12117954 PMC128161

[B41] Nett J. Lincoln L. Marchillo K. Massey R. Holoyda K. Hoff B. . (2007). Putative role of beta-1,3 glucans in *Candida albicans* biofilm resistance. Antimicrob. Agents Chemother. 51 (2), 510–520. doi: 10.1128/AAC.01056-06 17130296 PMC1797745

[B42] NIH (2005). Research on microbial biofilms. (Bethesda, Maryland, USA: National Institutes of Health (NIH)). Available at: https://grants.nih.gov/grants/guide/pa-files/pa-03-047.html.

[B43] Novick R. P. Geisinger E. (2008). Quorum sensing in staphylococci. Annu. Rev. Genet. 42, 541–564. doi: 10.1146/annurev.genet.42.110807.091640 18713030

[B44] O'Gara J. P. (2007). Ica and beyond: biofilm mechanisms and regulation in *Staphylococcus epidermidis* and *Staphylococcus aureus* . FEMS Microbiol. Lett. 270 (2), 179–188. doi: 10.1111/j.1574-6968.2007.00688.x 17419768

[B45] Ogata H. Goto S. Sato K. Fujibuchi W. Bono H. Kanehisa M. (1999). KEGG: Kyoto encyclopedia of genes and genomes. Nucleic Acids Res. 27 (1), 29–34. doi: 10.1093/nar/27.1.29 9847135 PMC148090

[B46] Otto M. (2008). Staphylococcal biofilms. Curr. Top. Microbiol. Immunol. 322, 207–228. doi: 10.1007/978-3-540-75418-3_10 18453278 PMC2777538

[B47] Parsek M. R. Singh P. K. (2003). Bacterial biofilms: an emerging link to disease pathogenesis. Annu. Rev. Microbiol. 57, 677–701. doi: 10.1146/annurev.micro.57.030502.090720 14527295

[B48] Parthasarathy A. Borrego E. J. Savka M. A. Dobson R. C. J. Hudson A. O. (2021). Amino acid-derived defense metabolites from plants: A potential source to facilitate novel antimicrobial development. J. Biol. Chem. 296, 100438. doi: 10.1016/j.jbc.2021.100438 33610552 PMC8024917

[B49] Porchezhian E. Dobriyal R. M. (2003). An overview on the advances of *Gymnema sylvestre*: chemistry, pharmacology and patents. Pharmazie 58 (1), 5–12. doi: 10.1002/chin.200319223 12622244

[B50] Rice K. C. Firek B. A. Nelson J. B. Yang S. J. Patton T. G. Bayles K. W. (2003). The *Staphylococcus aureus* cidAB operon: evaluation of its role in regulation of murein hydrolase activity and penicillin tolerance. J. Bacteriol. 185 (8), 2635–2643. doi: 10.1128/JB.185.8.2635-2643.2003 12670989 PMC152627

[B51] Rice K. C. Nelson J. B. Patton T. G. Yang S. J. Bayles K. W. (2005). Acetic acid induces expression of the *Staphylococcus aureus* cidABC and lrgAB murein hydrolase regulator operons. J. Bacteriol. 187 (3), 813–821. doi: 10.1128/JB.187.3.813-821.2005 15659658 PMC545714

[B52] Schilcher K. Horswill A. R. (2020). Staphylococcal biofilm development: Structure, regulation, and treatment strategies. Microbiol. Mol. Biol. Rev. 84 (3), e00026–19. doi: 10.1128/MMBR.00026-19 32792334 PMC7430342

[B53] Schlag S. Nerz C. Birkenstock T. A. Altenberend F. Gotz F. (2007). Inhibition of staphylococcal biofilm formation by nitrite. J. Bacteriol. 189 (21), 7911–7919. doi: 10.1128/JB.00598-07 17720780 PMC2168742

[B54] Sewell E. W. Brown E. D. (2014). Taking aim at wall teichoic acid synthesis: new biology and new leads for antibiotics. J. Antibiot (Tokyo) 67 (1), 43–51. doi: 10.1038/ja.2013.100 24169797

[B55] Sigoillot M. Brockhoff A. Neiers F. Poirier N. Belloir C. Legrand P. . (2018). The crystal structure of gurmarin, a sweet taste-suppressing protein: Identification of the amino acid residues essential for inhibition. Chem. Senses 43 (8), 635–643. doi: 10.1093/chemse/bjy054 30137256

[B56] Song W. Wang B. Sui L. Shi Y. Ren X. Wang X. . (2022). Tamarixetin attenuated the virulence of *Staphylococcus aureus* by directly targeting caseinolytic protease p. J. Nat. Prod. 85 (8), 1936–1944. doi: 10.1021/acs.jnatprod.2c00138 35833867

[B57] Taponen S. Pyorala S. (2009). Coagulase-negative staphylococci as cause of bovine mastitis- not so different from *Staphylococcus aureus* ? Vet. Microbiol. 134 (1-2), 29–36. doi: 10.1016/j.vetmic.2008.09.011 18977615

[B58] Tatusov R. L. Fedorova N. D. Jackson J. D. Jacobs A. R. Kiryutin B. Koonin E. V. . (2003). The COG database: an updated version includes eukaryotes. BMC Bioinf. 4, 41. doi: 10.1186/1471-2105-4-41 PMC222959 12969510

[B59] Vadyvaloo V. Otto M. (2005). Molecular genetics of *Staphylococcus epidermidis* biofilms on indwelling medical devices. Int. J. Artif. Organs 28 (11), 1069–1078. doi: 10.1177/039139880502801104 16353113

[B60] Vediyappan G. Dumontet V. Pelissier F. d'Enfert C. (2013). Gymnemic acids inhibit hyphal growth and virulence in *Candida albicans* . PloS One 8 (9), e74189. doi: 10.1371/journal.pone.0074189 24040201 PMC3770570

[B61] Vediyappan G. Rossignol T. d'Enfert C. (2010). Interaction of *Candida albicans* biofilms with antifungals: transcriptional response and binding of antifungals to beta-glucans. Antimicrob. Agents Chemother. 54 (5), 2096–2111. doi: 10.1128/AAC.01638-09 20194705 PMC2863626

[B62] Veerapandian R. Paudyal A. Chang A. Vediyappan G. (2020). Separation of bioactive small molecules, peptides from natural sources and proteins from microbes by preparative isoelectric focusing (IEF) method. J. Vis. Exp. 160, 10.2328. doi: 10.3791/61101 PMC8006215 32597857

[B63] Veerapandian R. Vediyappan G. (2019). Gymnemic acids inhibit adhesive nanofibrillar mediated *Streptococcus gordonii-candida albicans* mono-species and dual-species biofilms. Front. Microbiol. 10, 2328. doi: 10.3389/fmicb.2019.02328 31681200 PMC6797559

[B64] Vestby L. K. Gronseth T. Simm R. Nesse L. L. (2020). Bacterial biofilm and its role in the pathogenesis of disease. Antibiot. (Basel) 9 (2), 59. doi: 10.3390/antibiotics9020059 PMC7167820 32028684

[B65] Wang C. K. Colgrave M. L. Gustafson K. R. Ireland D. C. Goransson U. Craik D. J. (2008). Anti-HIV cyclotides from the Chinese medicinal herb *Viola yedoensis* . J. Nat. Prod. 71 (1), 47–52. doi: 10.1021/np070393g 18081258 PMC6327322

