# Supplementary material for: Inhibition of Staphylococcus aureus biofilm formation by gurmarin, a plant-derived cyclic peptide
Source: Front Cell Infect Microbiol. 2022 Oct 4;12:1017545. doi: 10.3389/fcimb.2022.1017545 (PMC9578378; doi:10.3389/fcimb.2022.1017545)
Supplement: Supplementary file 1 [file DataSheet_1.docx]

Supplementary Material

**Supplementary Figure 1.** **Inhibition of *S. aureus* biofilm by gurmarin.** The SH1000 strain of *S. aureus* was used to test if gurmarin (1 µg/ml) can inhibit biofilm growth in a 96-well microtiter plate for 24 h at 37^o^ C as mentioned in the Materials and methods, and found indeed it was the case. Crystal violet stained wells (**A**) and the absorbance value of the solubilized solution at OD _595 nm_ (**B**) are shown. *** *p* < 0.0002 paired Student’s *t*-test.

**Supplementary Figure 2.**

**Supplementary Figure 2. Gurmarin does not hemolyze red blood cells.** Tryptic soy agar (TSA) containing red blood cells (RBC) was used to spot test gurmarin for hemolytic activity. After 24-48 h incubation at 37^o^ C hemolytic activity was recorded. Gurmarin (5 µg in 5 µl spot) did not show hemolytic activity. Hemolytic activity (clear halo) around *S. aureus* (*Sa*) was seen while Triton-X 100 showed non-specific hemolysis and served as controls. A schematic with sample identity is shown on the right panel. Gur, gurmarin; *Sa*, *S. aureus* colony; Tri, Triton-X 100 (1%, 5 µl).
